# Exsolution-Engineered Perovskite Catalysts for Durable Plasma-Assisted Ammonia Synthesis

**DOI:** 10.3390/molecules31183254

**Published:** 2026-09-14

**Authors:** Sebastián Gámez, Abhyuday Chatterjee, Filippo Manaigo, Rony Snyders, Eric M. Gaigneaux

**Affiliations:** 1 Institute of Condensed Matter and Nanosciences (IMCN), Université Catholique de Louvain (UCLouvain), Place Louis Pasteur 1. L04.01.09, 1348 Louvain-la-Neuve, Belgium; sebastian.gamez@uclouvain.be; 2 Research Group ChIPS, Department of Chemistry, University of Mons, 20 Place du Parc, 7000 Mons, Belgium; rony.snyders@umons.ac.be (R.S.); 3 Materia Nova Research Center, 3 Avenue Copernic, 7000 Mons, Belgium; filippo.manaigo@materianova.be

**Keywords:** ammonia synthesis, perovskite exsolution, microwave plasma, sintering resistance, plasma catalysis

## Abstract

Ammonia is a cornerstone chemical for global food security and an emerging carbon-free energy carrier, yet its industrial synthesis via the Haber–Bosch process remains energy-intensive and carbon-emitting. Non-thermal plasma catalysis offers a promising decentralized alternative for nitrogen fixation under milder conditions, though the long-term structural stability of supported metal catalysts under harsh plasma environments remains a key open challenge. Plasma-assisted NH_3_ synthesis was investigated using exsolution-derived Co-La_2_O_3_/Al_2_O_3_ and Ni-La_2_O_3_/Al_2_O_3_ catalysts in a pulsed (1 kHz at 50% duty cycle) microwave reactor operating at sub-atmospheric pressure (2–6 Torr) and 2.45 GHz. The catalysts were prepared by reductive H_2_ treatment of LaCoO_3_ and LaNiO_3_ perovskite precursors, deposited onto Al_2_O_3_ pellets, triggering metal exsolution and generating metallic Co^0^ and Ni^0^ nanoparticles embedded within a La_2_O_3_/Al_2_O_3_ matrix. Catalytic performance was evaluated across a range of H_2_/N_2_ flow rates (200–400 cm^3^/min each) and microwave power inputs (0.6–0.7 kW average), using bare Al_2_O_3_ as a reference. Co-La_2_O_3_/Al_2_O_3_ outperformed both Ni-La_2_O_3_/Al_2_O_3_ and the Al_2_O_3_ reference under all tested conditions, reaching a maximum H_2_ conversion of 2.39% and a peak productivity of 20.6 μmol/g_cata_.h, attributed to the finer metal dispersion and smaller nanoparticle size achieved during Co exsolution from the LaCoO_3_ lattice. Post-reaction characterization by TEM, XRD and N_2_ physisorption confirmed the structural integrity of both spent catalysts. No bulk phase transformations were detected by XRD, while specific surface areas were retained above 93% of the initial one. Metals’ particle size slightly increased after plasma exposure, confirming the structural durability of catalysts. These results demonstrate that the exsolution mechanism confers meaningful sintering resistance under microwave plasma conditions, establishing exsolution-derived perovskite catalysts as a promising and durable platform for plasma-assisted nitrogen fixation.

## 1. Introduction

The global demand for ammonia (NH_3_) is expanding beyond its traditional role in the fertilizer industry to include its potential as a carbon-free energy carrier and hydrogen storage medium [1,2,3]. However, the conventional Haber–Bosch process, while highly efficient at scale, is responsible for approximately 2% of global energy consumption and 1.8% of CO_2_ emissions due to its reliance on fossil fuels and extreme operating conditions of high temperature and pressure [4,5]. Consequently, there is a burgeoning interest in Non-Thermal Plasma (NTP) technology as a sustainable, decentralized alternative [6]. Plasma-assisted synthesis allows for the activation of the highly stable N_2_ triple bond through electron impact rather than thermal energy, enabling the reaction to occur at ambient pressure and lower temperatures [6,7]. By integrating plasma catalysis, researchers are exploring synergistic effects that bypass the “Sabatier peak” limitations of traditional catalysts, aligning with the transition toward green ammonia powered by intermittent renewable energy sources [6,7,8].

This shift toward plasma-driven systems requires a fundamental change in how the reaction is understood mechanistically [9,10]. The conventional Haber–Bosch process operates via a surface-mediated Langmuir–Hinshelwood pathway, where the rate-determining step is the dissociative adsorption of the inert N_2_ molecule onto the catalyst active sites [9,10]. Due to the high bond dissociation energy of nitrogen (945 kJ⋅mol^−1^), extreme thermal energy is required to overcome the activation barrier, followed by stepwise hydrogenation to form NH_3_ [9,10,11]. In contrast, plasma-assisted synthesis shifts this activation to the gas phase through electron impact dissociation and vibrational excitation. In an NTP environment, high-energy electrons collide with N_2_ molecules, promoting them to vibrationally excited states (N_2(v)_). These “pre-activated” species exhibit significantly lower activation barriers for surface reactions, allowing the process to bypass the constraints of the Brønsted–Evans–Polanyi (BEP) relationship under non-equilibrium conditions [12,13,14].

Building on these theoretical advantages, the recent literature has explored transition-metal catalysts such as nickel (Ni), iron (Fe), and cobalt (Co) within Dielectric Barrier Discharge (DBD) reactors. For instance, Ni/Al_2_O_3_ catalysts have reached ammonia synthesis rates of approximately 471 μmol⋅g^−1^⋅h^−1^ at near-room temperature [12,15]. Similarly, Fe/Al_2_O_3_ leverages the high intrinsic activity of iron for nitrogen dissociation, while advanced Co-Ni/MOF-74 configurations have pushed productivity boundaries to 2608.70 μmol⋅g^−1^⋅h^−1^ [16,17]. Despite these promising rates, these systems face persistent challenges such as hydrogen poisoning, where H_2_ blocks active sites, and the “back-reaction”, where plasma electrons decompose the produced NH_3_ almost as quickly as it is formed [12,16,17]. Furthermore, energy efficiencies typically remain below 5 g-NH_3_/kWh in lab-scale setups, which is far from the threshold required for industrial viability [16,17,18].

Beyond kinetic limitations, the stability and structural integrity of these catalysts remain a critical bottleneck because the harsh environment of an electrical discharge differs fundamentally from thermal systems [18,19]. While traditional materials are designed for steady-state thermal stress, plasma-exposed catalysts are subjected to continuous ion bombardment and high-voltage stress, leading to physical sputtering and the redistribution of the active metal phase [19]. In DBD reactors, the formation of filamentary micro-discharges creates localized “hotspots” that trigger the sintering of metal nanoparticles and a significant loss of active surface area [19,20]. Additionally, reactive species can induce unwanted phase transformations, such as oxidation, while intense electric fields can cause the structural collapse of delicate architectures like MOFs or silica supports [21,22].

To address these stability and deactivation issues, this study investigates the use of LaNiO_3_ and LaCoO_3_ perovskite-type oxides as robust precursor materials [23,24]. Perovskites are a versatile class of inorganic compounds with the general formula ABO_3_, featuring a distinctive cubic crystal structure in which larger A-site cations (such as alkaline earth or rare-earth metals) and smaller B-site transition-metal cations are coordinated with oxygen anions [23,24,25,26]. These materials display high thermal and chemical stability, and excellent conductivity [23,24,25,26]. Owing to this structural flexibility, perovskite oxides have found broad application in electrocatalysis and energy-related technologies, including selective electrochemical sensing and advanced nanostructuring strategies for enhancing catalytic performance [27,28]. Through a controlled exsolution process, Ni and Co cations are migrated from the perovskite lattice to the surface, forming finely dispersed metallic nanoparticles that remain embedded within the parent lanthanum oxide matrix [25,26]. This unique submerged architecture provides a superior mechanical anchor that significantly enhances resistance to sintering and physical sputtering, even when subjected to intense plasma discharges. Beyond resilience, perovskites offer tunable defect chemistry and high lattice oxygen mobility to facilitate the stabilization of reactive intermediates. By utilizing these exsolved systems, we aim to leverage the strong metal–support interaction (SMSI) to maintain high ammonia productivity and long-term catalytic integrity under the aggressive non-equilibrium conditions of a plasma reactor.

## 2. Methodology

### 2.1. Synthesis of M-La_2_O_3_ (M = Ni, Co) Catalysts Supported on Alumina

**Scheme 1:**
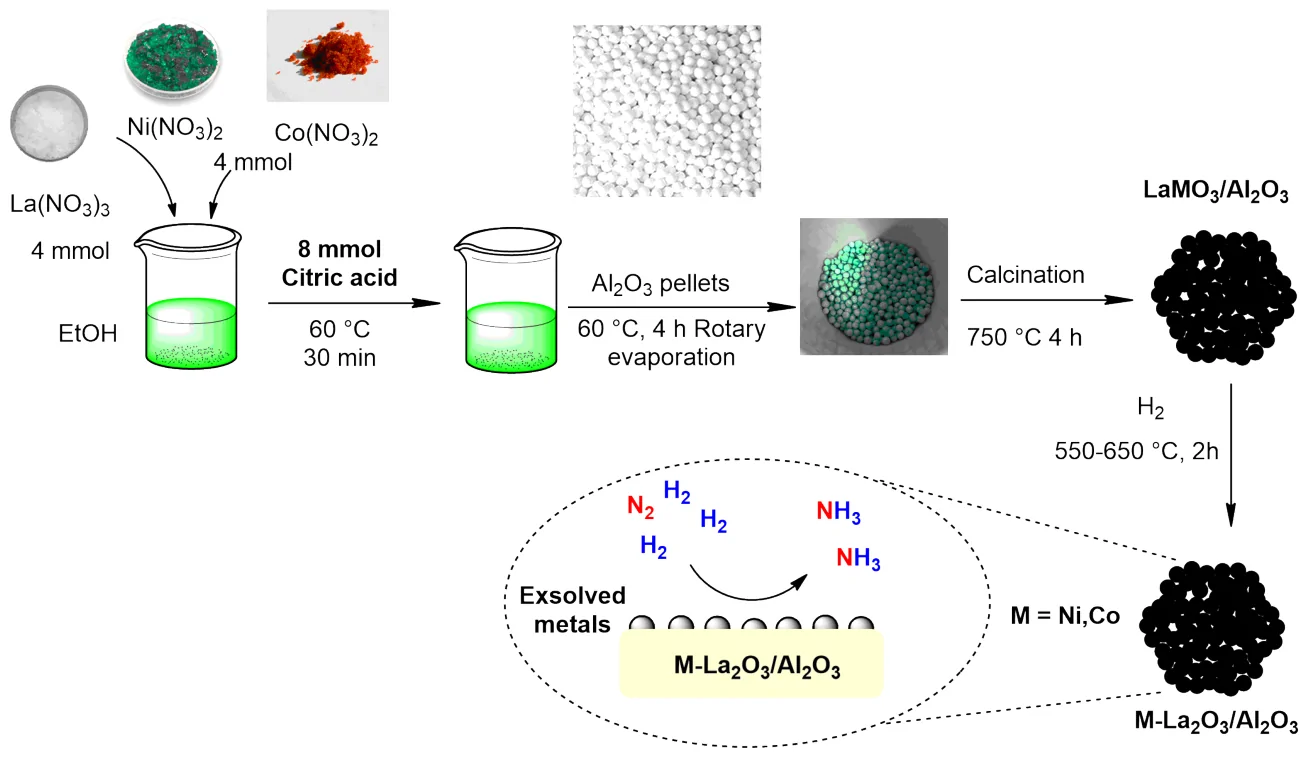
Synthesis of M-La_2_O_3_/Al_2_O_3_ (M = Co, Ni) catalysts for NH_3_ production under plasma conditions.

Catalysts in the form of pellets were synthesized using commercial alumina (Thermo Fischer Scientific, Waltham, MA, USA in the form of 4 mm pellets) as support to disperse the perovskites prior to exsolution. For this purpose, either 4 mmol of Ni(NO_3_)_2_.6H_2_O or Co(NO_3_)_2_.6H_2_O was dissolved along with 4 mmol of La(NO_3_)_3_.6H_2_O in 30 mL of ethanol and 10 mL of distilled H_2_O. Afterwards, 8 mmol of citric acid was added to the previous solution and magnetically stirred for 30 min at room temperature. Then, 1.00 g of alumina was added to the mixture, and the resulting suspension was agitated for 4 h at 60 °C. The solvent was then removed through rotary evaporation at 70 °C. The pellets were dried overnight at 110 °C, followed by calcination at 750 °C under air for 4 h with a heating ramp of 2 °C/min. Finally, perovskites over alumina were submitted to exsolution by flowing them in 20 mL/min of pure H_2_ at 550 °C for Ni samples and 650 °C for Co samples (according to H_2_-TPR analysis Figure S1) for 2 h using a heating ramp of 5 °C/min (Scheme 1).

### 2.2. Characterization Techniques

The textural properties of catalysts were evaluated via N_2_ physisorption at −196 °C using a Micromeritics Tristar 3000 instrument, Micromeritics Instrument Corporation, Norcross, GA, USA. Prior to measurement, approximately 100 mg of the sample underwent a degassing process at 200 °C for 12 h under vacuum (~0.1 mbar). The Brunauer–Emmett–Teller (BET) equation was applied to determine the specific surface area, considering the adsorption data within the 0.05 < P/P_0_ < 0.30 relative pressure range. Furthermore, the Barrett–Joyner–Halenda (BJH) method was employed to estimate pore size distributions and volumes from the desorption isotherms [29,30].

X-ray diffraction (XRD) patterns were acquired to verify the crystalline structure of nickel and cobalt perovskites. In addition, exsolved samples were analyzed to check the possibility of sintering after plasma catalytic conditions. Measurements were conducted on a Bruker-D8 Advance diffractometer (Bruker AXS GmbH, Karlsruhe, Germany) equipped with a Cu anode (Kα = 0.15418 nm) and Bragg–Brentano geometry, operated at 20 kV and 5 mA. Finely ground samples were mounted on polished silicon holders using a thin film of Nivea^®^ cream (Hamburg, Germany). Data collection spanned a 2θ range of 5° to 80° with a step size of 0.015° over a total duration of 26 min. Phase identification for both LaNiO_3_ and LaCoO_3_ systems was performed by comparison with the ICDD-PDF2-2004 database. The average crystallite sizes of the exsolved metallic nanoparticles (Ni^0^ and Co^0^) were determined using the Scherrer equation:

(1) $$d=\;\frac{K\lambda }{\beta Cos\theta }$$

In this expression:

- d represents the mean crystallite size.
- K is the dimensionless shape factor, taken as 0.98.
- λ denotes the X-ray wavelength (1.5418 Å).
- β is the line broadening at full width at half maximum (FWHM) after subtracting the instrumental broadening.

For the size calculation, the most intense reflections were selected: the (111) plane located at 2θ = 44.5° for metallic Ni, and the corresponding (111) plane at 2θ = 44.2° for metallic Co. To ensure accuracy and account for instrumental effects, high-purity Ni and Co powders with a known crystallite size of 100 nm were employed as reference standards for all calculations.

Metal nanoparticles’ size distribution before and after plasma catalytic conditions was assessed through high-resolution transmission electron microscopy (HRTEM) using a JEOL 2100 Plus UHR microscope operating at 200 kV (JEOL Ltd., Tokyo, Japan). The metallic surface area and the degree of dispersion for both Ni and Co exsolved systems were quantified via H_2_ chemisorption using a Microtrac Belcat II analyzer (MicrotracBEL Corp., Osaka, Japan). In a typical measurement, approximately 100 mg of the sample was pre-conditioned at 150 °C for 3 h under a constant He flow (50 mL/min) to remove physisorbed species. Following this step, an in situ reduction was performed by heating the samples to 600 °C (at a heating rate of 10 °C/min) under a reactive mixture of H_2_ and He (20/30 mL/min, respectively) for 1 h. After reduction, the system was flushed and cooled to 50 °C under an Ar atmosphere (50 mL/min). The chemisorption analysis was then conducted by injecting calibrated pulses (0.5 mL) of a 0.5 *v*/*v*% H_2_/Ar mixture until saturation was reached. The quantity of H_2_ consumed was recorded using a thermal conductivity detector (TCD). To derive the metallic surface area and dispersion, an adsorption stoichiometry of M:H = 1 (where M = Ni or Co) was assumed, following established protocols for these transition metals [31].

### 2.3. Catalytic Tests

A 2.45 GHz microwave magnetron surfaguide was used to generate the plasma inside a quartz tube with an inner diameter of 14 mm. The quartz tube was cooled to a temperature of 16 °C with silicon oil flowing through another larger, concentric, quartz tube. The microwave injection was pulsed at 1 kHz at 50% duty cycle. A constant feed of N_2_ and H_2_ was provided through two independent mass flow controllers, and the system was kept, via a dry pump, in a pressure range between 2 and 6 Torr, depending on the flow rate and on the presence of catalysts. During the experiments, the N_2_ flow was set at 200 and 400 cm^3^/min, and the H_2_ flow at 200 and 400 cm^3^/min. The catalyst pellets were held downstream within a cylindrical stainless steel holder at approximately 8 cm from the surfaguide. The cylinder had two ceramic grids that allowed the gas to reach and interact with the catalysts. The typical amount of catalysts used during the tests was 5 g. Forwarded and reflected microwave power were monitored via a directional coupler for every tested condition, and the net absorbed power (forwarded minus reflected) was used to calculate the energy-normalized NH_3_ yield reported in Section 3.

H_2_ and NH_3_ concentrations in the reactor outlet were quantified using a micro-GC equipped with a thermal conductivity detector (TCD). The sampled gas was diluted with He to reach an injection pressure of 2 bar, and the reactor exhaust was diluted with N_2_ upstream of the vacuum pump to ensure safe handling of the H_2_-containing stream. Because of these dilutions, absolute NH_3_ concentration could not be obtained directly; instead, the system was calibrated using a series of known H_2_:NH_3_:N_2_ gas mixtures (H_2_ = 5, NH_3_ = 1–5, N_2_ balance), confirming a linear NH_3_/H_2_ peak-area response across the calibration range. This calibration was used to convert the measured NH_3_ peak area into an equivalent NH_3_ concentration (Equation (2)), referenced to the unconverted-H_2_ signal to determine the NH_3_ fraction and H_2_ conversion factor, following the reaction stoichiometry N_2_ + 3H_2_ → 2NH_3_ (Equation (3)). The N_2_ signal was excluded from the conversion calculation, as the N_2_ dilution described above alters the apparent N_2_ fraction at the sampling point. NH_3_ productivity was normalized to the catalyst mass and expressed on a per-hour basis (Equation (4)).

C_NH3_ (%) = 4.8708 × 10^−5^ × A_NH3_ − 2.6398 × 10^−6^ (2)

X(NH_3_) = C_NH3_/[C_NH3_ + (2/3)·C_H2_] (3)

Productivity (μmol g_cata_^−1^ h^−1^) = (C_NH3_/100) × F_total_ × (10^6^/V_m_) × 60/m_cata_ (4)

where A_NH3_ is the integrated GC peak area of NH_3_; C_H2_ and C_NH3_ are the measured downstream mole fractions (%) of H_2_ and NH_3_, respectively; F_total_ is the total inlet gas flow rate (cm^3^/min); V_m_ = 22,414 cm^3^ mol^−1^ is the standard molar volume of an ideal gas; and m_cata_ = 5 g is the catalyst mass. A representative worked example is provided in the Supporting Information.

Each operating point was held until the GC signal was stable before sampling, and each condition was tested for approximately 10 min in triplicate (n = 3 independent measurements), corresponding to a total plasma exposure time of approximately 180 min per catalyst across the six flow/power conditions tested. Error bars represent the standard deviation (±SD) of these replicate measurements. The H_2_:N_2_ ratio, total flow rate, catalyst mass, and estimated residence time for each condition are summarized in Table S2 of the Supporting Information.

## 3. Results and Discussion

**Figure 1:**
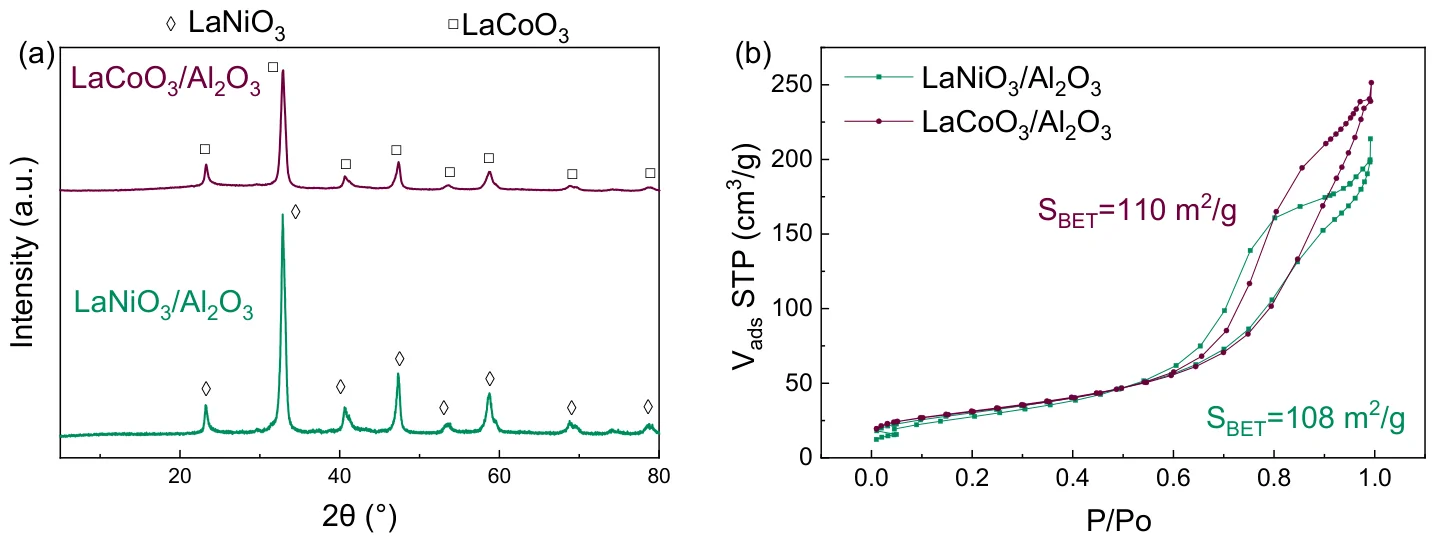
(**a**) Diffractograms of perovskites LaMO_3_/Al_2_O_3_ deposited on alumina surface; (**b**) N_2_ physisorption isotherms of LaMO_3_/Al_2_O_3_ (M = Co, Ni).

The crystalline structure of the synthesized catalysts was verified through X-ray diffraction. Figure 1a displays the diffractograms for the LaNiO_3_ and LaCoO_3_ phases supported on γAl_2_O_3_ pellets. In the case of the nickel perovskite, the sample exhibits distinct diffraction reflections at 2θ values of 23.3°, 32.9°, 40.6°, 41.2°, 47.4°, 53.7°, 58.8°, 69.7°, and 78.9°. This diffraction pattern is consistent with the rhombohedral perovskite structure of LaNiO_3_, in excellent agreement with the ICDD-PDF 79-2451 standard [32]. These peaks are indexed to the (101), (110), (021), (003), (202), (211), (122), (220), and (312) planes, respectively. As regards LaCoO_3_ perovskite, it shows characteristic reflections at approximately 23.2°, 32.9°, 33.3°, 40.7°, 47.5°, 53.3°, 59.0°, and 69.1°. These signals correspond to the (012), (110), (104), (202), (024), (116), (214), and (300) Miller indices, confirming the formation of the rhombohedral LaCoO_3_ phase (ICDD-PDF 48-0123) [33]. These results confirm the successful integration of the perovskite phase onto the alumina pellets without significant structural degradation. Alumina characterization is displayed in Figure S2a to show the structure of the reference material employed in this work.

The textural characteristics of the bare support and the perovskite-loaded catalysts were evaluated via N_2_ physisorption. The bare γAl_2_O_3_ pellets exhibited a characteristic Type IV isotherm with a well-defined hysteresis loop with parallel branches, a profile typical of open cylindrical mesoporous systems (Figure S2b). It is important to note that the porosity in these pellets was predominantly interparticular, arising from the strategic packing of primary alumina grains rather than intrinsic intraparticle channels. The pristine support possessed a high specific surface area of 232 m^2^/g and a total pore volume of 0.68 cm^3^/g, providing an extensive network of interparticle voids for the dispersion of the active phases.

Upon the deposition of the LaNiO_3_ and LaCoO_3_ perovskite phases, a notable decrease in the textural parameters was observed. The specific surface area decreased to 108 m^2^/g and 110 m^2^/g for the nickel and cobalt systems, respectively, while the total pore volume was reduced to 0.3 cm^3^/g in both cases (Table 1). This significant reduction was attributed to a combination of the mass dilution effect due to the high density of the crystalline perovskite and the physical occupancy of the interparticle voids by the LaMO_3_ (M = Ni, Co) nanocrystals, which effectively reduced the available space between the alumina grains.

Interestingly, despite the substantial drop in specific surface area and volume, the average pore size remained remarkably constant around a value of 8.5 nm. In the context of interparticle porosity, the stability of this dimension suggests that the perovskite phase does not form a conformal, uniform coating over the individual alumina particles, as such a layer would progressively narrow the gaps between them. Instead, the results indicate that the LaMO_3_ (M = Ni, Co) crystallites likely promote the complete occlusion of a fraction of the interparticle voids while leaving the remaining accessible network unobstructed. This selective filling mechanism accounts for the loss in total volume while preserving the characteristic diameter of the surviving intergranular spaces.

**Figure 2:**
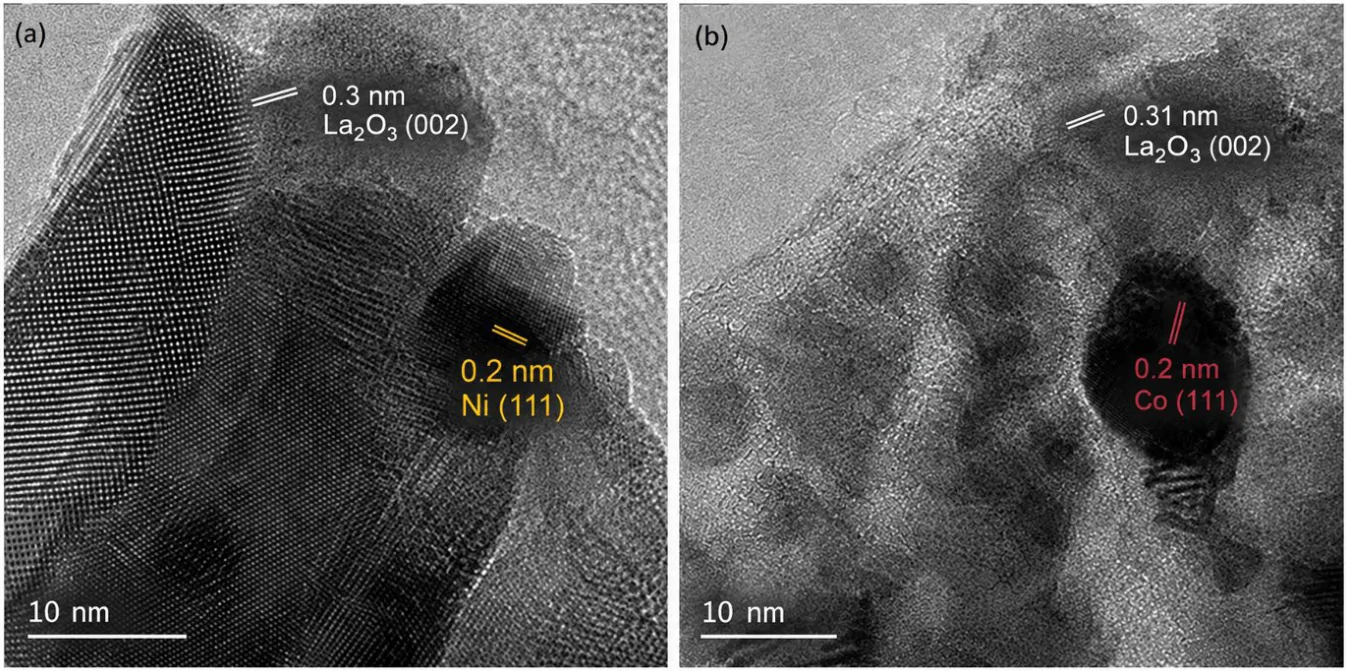
HR-TEM images of (**a**) Ni-La_2_O_3_/Al_2_O_3_ and (**b**) Co-La_2_O_3_/Al_2_O_3._

To directly evidence Co and Ni exsolution from perovskite matrix, high-resolution transmission electron microscopy (HR-TEM) was performed on both catalysts. The representative lattice fringe analyses for Ni-La_2_O_3_/Al_2_O_3_ and Co-La_2_O_3_/Al_2_O_3_ are shown in Figure 2a and Figure 2b, respectively. In both cases highly crystalline lattice fringes are clearly visible at the interface between the metallic nanoparticles and the surrounding oxide phase. The nanoparticles reveal well-defined lattice fringes with a d-spacing of 0.2 nm, characteristic of the (111) plane of metallic nanoparticles. They are intimately encapsulated and anchored within the La_2_O_3_ support, which exhibits characteristic lattice fringes with a d-spacing of 0.31 nm assigned to the (002) plane.

These HR-TEM lattice measurements provide definitive microscopic evidence of the exsolution phenomenon occurring during H_2_ reduction of the precursor perovskites. The physical embedding of the metallic Ni^0^ and Co^0^ nanoparticles within La_2_O_3_ lattice generates a strong metal–support interaction (SMSI) that effectively locks the active phase in place.

## 4. Catalytic Tests

**Figure 3:**
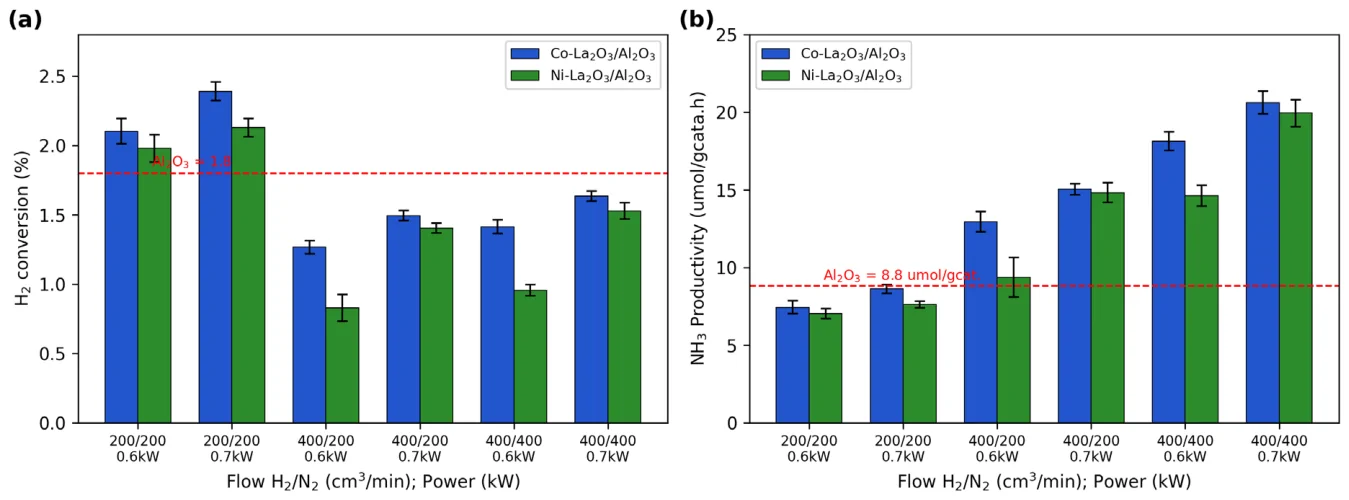
Catalytic performance of M-La_2_O_3_-Al_2_O_3_ (M = Co, Ni) catalysts under different plasma conditions: (**a**) H_2_ conversion into NH_3_; (**b**) ammonia productivity.

The catalysts evaluated in this work were obtained by reductive H_2_ treatment of LaNiO_3_ and LaCoO_3_ perovskite precursors, a process designed to trigger the exsolution of metallic cations and generate metallic Ni^0^ and Co^0^ nanoparticles uniformly anchored on a La_2_O_3_ matrix Both resulting materials demonstrated measurable and reproducible catalytic activity for plasma-assisted NH_3_ synthesis, with the Co-La_2_O_3_/Al_2_O_3_ system outperforming its Ni counterpart across all tested flow and power conditions. The exsolved Co-La_2_O_3_/Al_2_O_3_ reached a maximum H_2_-to-NH_3_ conversion factor of 2.39% at 200/200 cm^3^/min (H_2_/N_2_) and 0.7 kW, compared to 2.13% for the Ni-La_2_O_3_/Al_2_O_3_ catalyst under identical conditions. This gap, while modest in absolute terms, was consistent across all tested conditions (Figure 3).

This performance difference is likely rooted in the intrinsic reducibility and exsolution behavior of metallic cations [34]. Co is among the most readily exsolvable transition metals from perovskite lattices under H_2_ reduction, typically yielding finer metal dispersion than Ni under equivalent conditions [33,34]. This is consistent with our H_2_ chemisorption results, where Co dispersion (1.8%) was higher than Ni dispersion (1.3%), and with the smaller mean Co nanoparticle size measured by TEM. We further hypothesize that the basic character of the La_2_O_3_ support matrix, preserved and likely enhanced after perovskite decomposition, could contribute to N_2_ activation and NHx intermediate stabilization, by analogy with mechanisms proposed for other basic oxide supports in plasma-catalytic NH_3_ synthesis [12].

Both catalysts surpassed the bare Al_2_O_3_ reference (XH_2_ = 1.8%, productivity = 8.8 μmol g_cata_.h) under most tested conditions, confirming that the exsolved metal nanoparticles contribute a genuine catalytic effect rather than merely modifying the plasma discharge through dielectric packing. When normalized to the 5 g catalyst bed, NH_3_ productivities ranged from approximately 7 to 21 μmol g_cata_.h, reaching peak values of 20.6 and 19.9 μmol g_cata_.h for Co-La_2_O_3_/Al_2_O_3_ and Ni-La_2_O_3_/Al_2_O_3_, respectively, at 400/400 cm^3^/min and 0.7 kW. These results also reveal a clear and physically meaningful trade-off between conversion efficiency and absolute throughput: lower total flow rates (400 cm^3^/min, H_2_/N_2_ = 1:1) maximize XH_2_ by extending the residence time of reactive plasma species at the catalyst surface, while higher flow rates (800 cm^3^/min) increase the molar flux of NH_3_ produced per unit time despite the drop in per-molecule conversion. This behavior is well established in plasma-catalytic packed-bed systems and underscores the importance of reporting both metrics when comparing catalyst performance across studies [12].

Placed within the broader literature on plasma-catalytic NH_3_ synthesis, the productivities reported here remain below the state-of-the-art values achieved in optimized DBD systems. Wang et al. reported a maximum NH_3_ synthesis rate of 471 μmol g_cata_.h using Ni/Al_2_O_3_ in a near-room-temperature DBD reactor [12]. Table 2 broadens the comparison and shows that several Ni- and Co-based DBD systems reported in the literature exhibit energy-normalized NH_3_ yields in the range of 0.4–1.15 g NH_3_ kWh^−1^ [35,36]. At the peak productivity condition of this work (20.6 μmol g_cata^−1^ h^−1^, 5 g catalyst, 0.7 kW nominal, 0.575 kW absorbed), the corresponding energy yield was approximately 3.05 mg NH_3_ kWh^−1^. Forwarded and reflected microwave power were monitored for every tested condition via a directional coupler; across the twelve catalyst-condition combinations tested, the net absorbed power ranged from 63% to 82% of the nominal generator power. Even using the more conservative, absorbed-power-based energy yield, this value remains markedly lower than the literature values in Table 2, which are reported on a nominal-power basis in their original sources. This gap is consistent with the fundamental differences in reactor design and operating regime summarized in Table 2, including the sub-atmospheric pressure and microwave power coupling used in this work, rather than necessarily reflecting a difference in intrinsic catalyst quality. However, several factors must be carefully weighed before this gap is interpreted as a shortcoming of the exsolution approach. First, the plasma configuration used here, a surface-wave microwave discharge (surfaguide), differs fundamentally from DBD in terms of power coupling efficiency, reactive species density, and plasma-catalyst contact geometry; direct productivity comparisons across plasma types carry significant uncertainty and are not yet standardized in the field. Second, and more importantly, the strategic value of the exsolution preparation route does not reside primarily in maximizing initial productivity figures, but in addressing the long-term stability challenge that has plagued supported metal catalysts in plasma environments. Nanoparticles formed via exsolution are well anchored within the parent oxide lattice, conferring increased resistance to sintering compared to conventional deposition methods [37]. This strong metal–support interaction, arising from the embedded structure of exsolved particles, is generally associated with smaller and more uniformly distributed nanoparticles than those obtained by impregnation. In the context of plasma catalysis, where local heating at discharge filaments and prolonged exposure to reactive radical species can accelerate catalyst deactivation, this structural robustness represents a significant practical advantage that is not captured by single-point productivity measurements. Accordingly, the results presented here should be understood as a proof-of-concept demonstrating the feasibility and promise of exsolution-derived perovskite catalysts for plasma-assisted NH_3_ synthesis, with further optimization of particle size, reduction conditions, and reactor-plasma coupling expected to substantially improve the absolute productivity toward values competitive with those reported in the DBD literature.

## 5. Post-Characterization

**Figure 4:**
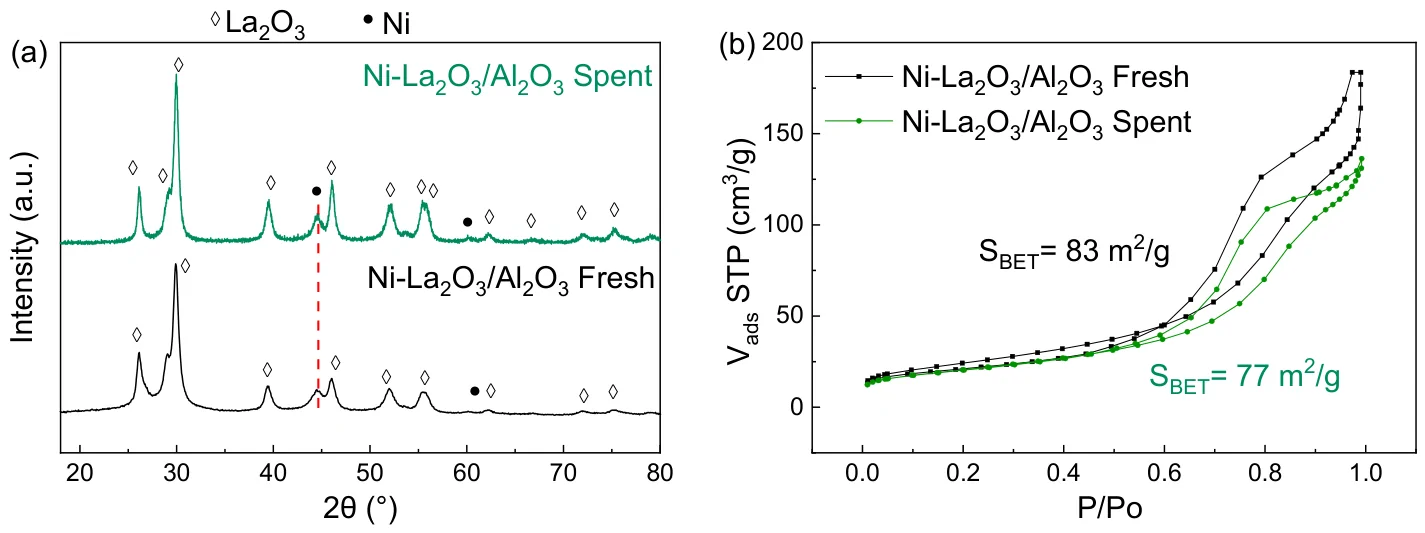
(**a**) Diffractograms and (**b**) N_2_ physisorption isotherms of Ni-La_2_O_3_/Al_2_O_3_ catalyst before and after plasma catalytic reaction.

**Figure 5:**
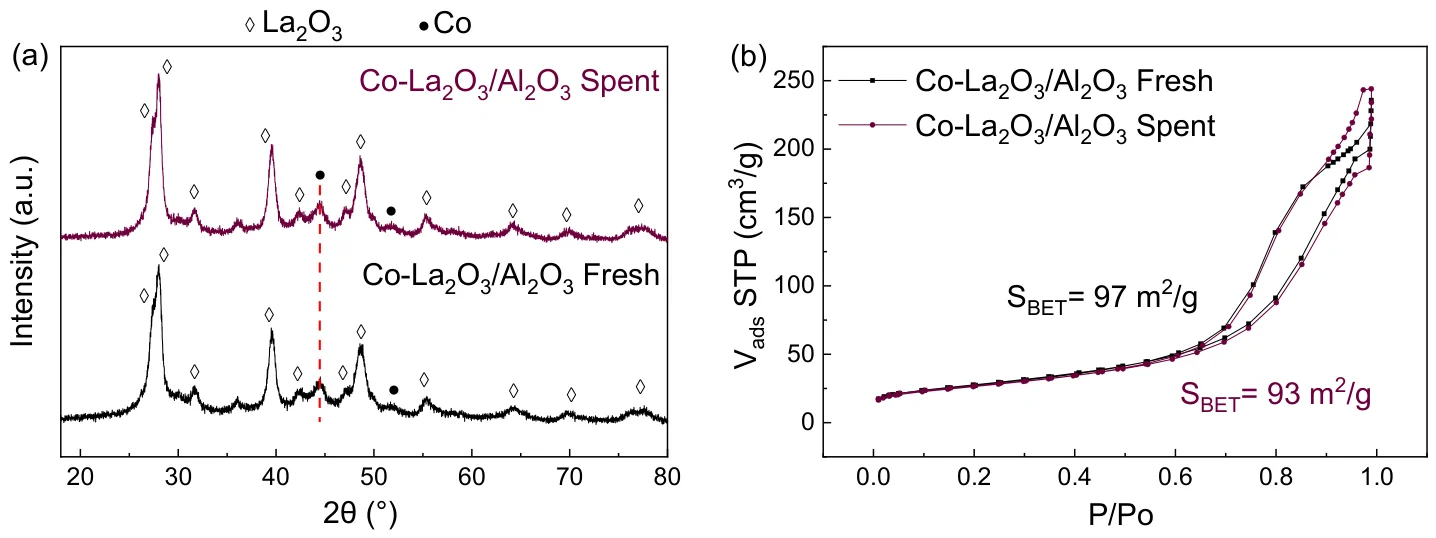
(**a**) Diffractograms and (**b**) N_2_ physisorption isotherms of Co-La_2_O_3_/Al_2_O_3_ catalyst before and after plasma catalytic reaction.

For clarity, the specific surface area values discussed in this section refer to the reduced, exsolved catalysts (denoted “Fresh” in Figure 4 and Figure 5), obtained after the H_2_ reductive pre-treatment described in the Section 2 distinct from the as-synthesized perovskite/Al_2_O_3_ precursor values reported in Table 1, which were measured prior to H_2_ reduction. The corresponding “Spent” values refer to the same exsolved catalysts after plasma-catalytic testing.

It should be noted that the durability discussed throughout this work refers specifically to the structural and textural resilience of the exsolved nanoparticle architecture, substantiated by ex situ, before/after characterization (XRD, N_2_ physisorption, TEM) comparing fresh and spent catalysts under plasma exposure, rather than by continuous time-on-stream monitoring of catalytic activity. To assess the structural and textural stability of the exsolution-derived catalysts under the demanding conditions imposed by the microwave plasma reactor, both spent materials were subjected to XRD analysis and N_2_ physisorption. Despite the highly reactive environment generated by the microwave discharge with continuous exposure to atomic nitrogen, hydrogen radicals, and vibrationally excited N_2_ species, both catalysts retained their essential structural integrity. These conditions, combined with the large catalyst bed mass of 5 g, represent a stringent stability test for any nanostructured catalyst system.

The XRD patterns of both spent catalysts revealed that the primary crystalline phases were preserved after plasma exposure (Figure 4). In both Ni-La_2_O_3_/Al_2_O_3_ and Co-La_2_O_3_/Al_2_O_3_, the characteristic reflections of the La_2_O_3_ matrix and the alumina support remained intact, and the metallic Ni^0^ and Co^0^ phases (generated during the H_2_ reductive exsolution pre-treatment) were still identifiable in the diffractograms of the spent materials. No new secondary phases bulk phases were detected. It should be noted that XRD is primarily sensitive to bulk crystalline phases; therefore, the absence of new diffraction reflections cannot rule out the formation of thin, poorly crystalline, or sub-detection-limit surface oxide or nitride layers. Accordingly, the conclusions drawn here regarding chemical stability are limited to the bulk crystalline structure of the catalysts.

The Scherrer analysis showed a moderate growth in mean crystallite size for both materials: from 15 to 19 nm for Ni-La_2_O_3_/Al_2_O_3_ and from 13 to 15 nm for Co-La_2_O_3_/Al_2_O_3_ (Table S1). While this indicates that some degree of sintering or particle coarsening occurred over the course of the experiments, the absolute magnitude of crystallite growth is modest in both cases, and notably more contained in the cobalt-based system, which is consistent with the higher catalytic performance sustained by Co-La_2_O_3_/Al_2_O_3_ throughout the catalytic tests (Figure 3). In addition, a counterexample was also assessed where Cobalt nanoparticles were deposited over alumina pellets through classical wet impregnation, at the same nominal Co loading (~20 wt%) and subjected to identical reductive pre-treatment (650 °C, 2 h under H_2_) and plasma-catalytic conditions as the exsolved Co-La_2_O_3_/Al_2_O_3_ catalyst. Table S1 shows that after plasma exposure a dramatic increase in crystallite size is evidenced (from not detected up to 64 nm), which confirms the benefits of the exsolution technique as a tool to keep the active site immobilized during plasma catalytic applications.

The N_2_ physisorption data provided a complementary and equally telling picture of the structural resilience of these materials (Figure 5). The specific surface areas of the spent catalysts (i.e., 77 m^2^/g for Ni-La_2_O_3_/Al_2_O_3_ and 93 m^2^/g for Co-La_2_O_3_/Al_2_O_3_) represented losses of only 7% and 4% relative to the fresh materials, respectively (Table S1). Pore volume and mean pore diameter were essentially unaffected by the plasma treatment in both cases, with Co-La_2_O_3_/Al_2_O_3_ retaining 96% of its original pore volume (0.27 vs. 0.28 cm^3^/g) and Ni-La_2_O_3_/Al_2_O_3_ retaining 90% (0.19 vs. 0.21 cm^3^/g). The shapes of the N_2_ adsorption–desorption isotherms remained unchanged between fresh and spent samples for both materials, confirming that the mesoporous texture of the Al_2_O_3_ framework, which served as the macroscopic support for the exsolution-derived La_2_O_3_/metal phase, was not disrupted by prolonged plasma exposure. Together, these findings demonstrate that the porous architecture built during the reductive exsolution process is effectively preserved under the microwave plasma conditions used in this study, with no evidence of pore collapse, extensive blockage by surface deposits, or restructuring of the support network.

All in all, the post-characterization data paint a coherent and encouraging picture of the structural stability of the exsolution-derived catalysts. The Co-La_2_O_3_/Al_2_O_3_ material, which exhibited the highest NH_3_ productivity among the tested catalysts, also displayed the greatest resistance to sintering and surface area loss with a crystallite size increase of only ~15% and a surface area retention of 96%. This parallel between superior activity and superior stability is not coincidental. In fact, it reflects the fundamental advantage of the exsolution preparation strategy, in which the metallic nanoparticles are not merely deposited onto the support surface but emerge from within the oxide lattice and remain partially embedded in it. This effect, well documented in the exsolution literature, generates a strong metal–support interaction that mechanically and chemically anchors the nanoparticles against lateral migration and coalescence, even under thermally and chemically aggressive conditions. The data reported here demonstrate that this sintering resistance is not merely a property of model systems or idealized thermal conditions but extends to the highly non-equilibrium environment of a low-pressure microwave plasma, which is a finding with direct practical implications for the design of structurally resilient catalysts in plasma-assisted NH_3_ synthesis.

**Figure 6:**
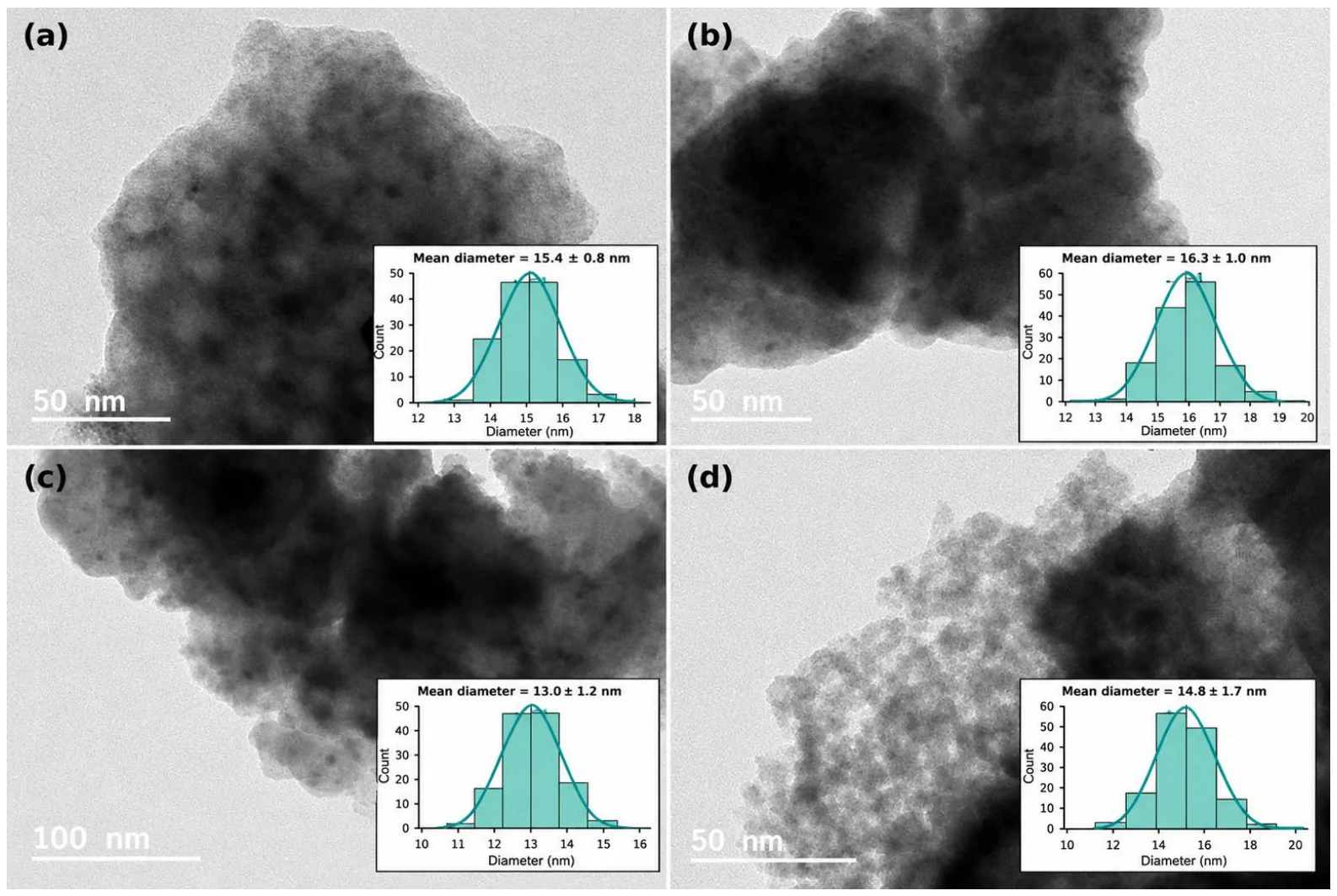
TEM images of: (**a**) fresh Ni-La_2_O_3_/Al_2_O_3_ (before catalytic test); (**b**) spent Ni-La_2_O_3_/Al_2_O_3_ (after catalytic test); (**c**) fresh Co-La_2_O_3_/Al_2_O_3_ (before catalytic test); (**d**) spent Co-La_2_O_3_/Al_2_O_3_ (after catalytic test).

To evaluate the morphological features, metal nanoparticle dispersion, and structural stability of the exsolved catalysts before and after exposure to microwave plasma, transmission electron microscopy (TEM) analysis was performed (Figure 6). As shown in Figure 6a,c, the H_2_ reductive pre-treatment successfully induced metal exsolution from the parent perovskite lattices (LaNiO_3_ and LaCoO_3_). In both fresh catalysts, nanoscale metallic particles (Ni^0^ and Co^0^) are uniformly distributed and firmly anchored across the oxide support matrix. In the fresh Ni-La_2_O_3_/Al_2_O_3_ sample (Figure 6a), the Ni nanoparticles display a well-defined spherical morphology with a mean diameter of 15.4 ± 0.8 nm. By comparison, the fresh Co-La_2_O_3_/Al_2_O_3_ catalyst (Figure 6c) exhibits smaller Co nanoparticles, with an average diameter of 13.0 ± 1.2 nm. The lower particle size in Co catalyst yielded a better catalytic performance during plasma-assisted NH_3_ synthesis.

Following the plasma-catalytic reaction, the spent catalysts were examined to assess their resistance to sintering under microwave-plasma conditions (Figure 6b,d). For spent Ni-La_2_O_3_/Al_2_O_3_ (Figure 5b), the mean particle size increased only slightly to 16.3 ± 1.0 nm. Likewise, spent Co-La_2_O_3_/Al_2_O_3_ (Figure 6d) showed a marginal increase in particle size, to 14.8 ± 1.7 nm. In both exsolved systems, the particle-size distributions remained relatively narrow, with no evidence of significant agglomeration or severe thermal coarsening, despite exposure to energetic ion bombardment, local micro-discharges, and reactive radical species within the plasma reactor. These microscopic observations are in agreement with data observed during XRD analyses.

The overall stability profile observed for both Co-La_2_O_3_/Al_2_O_3_ and Ni-La_2_O_3_/Al_2_O_3_ compares favorably with what would be expected from conventional impregnated catalysts of similar metal loading subjected to equivalent plasma conditions. Wet-impregnated Ni or Co nanoparticles on alumina are known to undergo significant agglomeration and surface area loss even under mild thermal treatments, with sintering accelerated by the presence of reactive surface species such as those generated in plasma environments. In contrast, the minimal textural degradation observed here, particularly for the Co-based system, provides evidence that the embedded nanoparticle morphology formed by exsolution translates into a real and measurable structural resilience advantage in an operationally relevant plasma reactor.

## 6. Conclusions

This work demonstrated the feasibility of exsolution-derived perovskite catalysts as active and structurally stable materials for plasma-assisted NH_3_ synthesis in a microwave reactor. By subjecting LaNiO_3_ and LaCoO_3_ precursors deposited over Al_2_O_3_ pellets to reductive H_2_ treatment, metallic Ni^0^ and Co^0^ nanoparticles were successfully generated in situ on a La_2_O_3_/Al_2_O_3_ matrix through exsolution, yielding catalyst systems with well-anchored, embedded nanoparticles that were intrinsically resistant to sintering.

Both exsolution-derived catalysts outperformed the bare Al_2_O_3_ reference under most tested conditions, confirming that the metallic nanoparticles contribute a genuine catalytic effect to the plasma-surface interaction rather than acting merely as a dielectric packing material. Co-La_2_O_3_/Al_2_O_3_ consistently achieved higher NH_3_ conversion and productivity than its Ni-based counterpart across all flow rates and power settings, reaching a maximum H_2_ conversion of 2.39% and a peak productivity of 20.6 μmol/g_cata_.h at 400/400 cm^3^/min and 0.7 kW. This performance advantage is attributed to the finer dispersion and smaller particle size of exsolved Co^0^ nanoparticles relative to Ni^0^ (Figure 6), consistent with the greater reducibility of Co^3+^ in the LaCoO_3_ lattice.

Post-reaction characterization confirmed the structural resilience of both catalysts after plasma exposure. XRD analysis revealed no evidence, within the bulk-sensitivity limits of this technique, of phase transformations, nitridation, or re-oxidation in either spent material, while N_2_ physisorption measurements showed surface area retentions of 93% and 96% for Ni/La_2_O_3_ and Co/La_2_O_3_, respectively, with negligible changes in pore volume and mean pore diameter. The moderate crystallite growth observed by Scherrer analysis was more pronounced in the Ni-based system (15 to 19 nm) than in the Co-based one (13 to 15 nm). The same trend was observed in TEM analyses where particle size barely increased after plasma exposure. These results are consistent with the activity trends and underscore the superior stability of the cobalt system. In addition, they provide direct experimental evidence that the exsolution mechanism to produce embedded metallic nanoparticles confers meaningful sintering resistance under the non-equilibrium conditions of a low-pressure microwave plasma, extending its demonstrated benefits beyond conventional thermal catalytic environments.

While the absolute NH_3_ productivities reported here remain below state-of-the-art values achieved in optimized DBD systems, the comparison must be interpreted in light of the fundamental differences in plasma configuration, catalyst bed geometry, and critically, the long-term structural resilience perspective that motivates the exsolution approach. The results presented in this study establish a solid proof-of-concept and open a clear path for further development: systematic optimization of exsolution conditions, nanoparticle size, and metal loading, combined with reactor-level engineering of the plasma-catalyst contact zone, are expected to substantially narrow the productivity gap while preserving the structural stability that constitutes the central merit of this catalyst design strategy.

## Figures and Tables

**Table 1:** Textural properties of perovskites on alumina.

Sample	Specific Surface Area (m^2^/g) ^a^	Pore Volume (cm^3^/g) ^b^	Mean Pore Diameter (nm) ^b^
Al_2_O_3_	232	0.68	8.5
LaNiO_3_/Al_2_O_3_	108	0.27	8.6
LaCoO_3_/Al_2_O_3_	110	0.32	8.4

^a^ BET method; ^b^ BJH method.

**Table 2:** Comparison of operating parameters and performance metrics between this work and representative literature plasma-catalytic NH_3_ synthesis systems.

System	Reactor/Conditions	Catalyst	NH_3_ Productivity	Energy Yield
This work	MW surfaguide, 2–6 Torr, RT, 400–800 cm^3^/min	Co-La_2_O_3_/Al_2_O_3_ (~20 wt%)	20.6 μmol gcata^−1^ h^−1^	~3.05 mg NH_3_ kWh^−1^ (absorbed power)
Wang et al. [12]	DBD, 1 atm, ~35 °C, 56 mL/min, N_2_:H_2_ = 1:2, 2 g cat.	Ni/Al_2_O_3_ (5 wt%)	Up to ~471 μmol gcata^−1^ h^−1^	Not reported
Zhou et al. [35]	DBD, 1 atm	Ni/Al_2_O_3_	Not reported	0.47–1.15 g NH_3_ kWh^−1^
Veng et al. [36]	Membrane DBD, 1 atm	Co/Al_2_O_3_ (5 wt%)	Not reported	0.82 g NH_3_ kWh^−1^

## Data Availability

The original contributions presented in this study are included in the article/Supplementary Material. Further inquiries can be directed to the corresponding author.

## Supplementary Materials

The following supporting information can be downloaded at: https://www.mdpi.com/article/10.3390/molecules31183254/s1, Figure S1. H_2_-TPR profile of LaNiO_3_/Al_2_O_3_ and LaCoO_3_/Al_2_O_3_. Figure S2. (a) XRD patterns and (b) N_2_ physisorption isotherm of alumina pellets. Table S1. Textural properties of Fresh and Spent catalysts. Figure S3. BJH pore size distributions (dV/dd_p_) of the bare Al_2_O_3_ support and the LaNiO_3_/Al_2_O_3_ and LaCoO_3_/Al_2_O_3_ precursor materials reported in Table 1. Figure S4. BJH pore size distributions (dV/dd_p_) of (a) Ni-La_2_O_3_/Al_2_O_3_ and (b) Co-La_2_O_3_/Al_2_O_3_ catalysts, Fresh and Spent, corresponding to the mean pore diameters reported in Table S1. Table S2. Testing conditions and estimated residence time for each flow/power condition.
