# Supplementary material for: Exsolution-Engineered Perovskite Catalysts for Durable Plasma-Assisted Ammonia Synthesis
Source: Molecules. 2026 Sep 14;31(18):3254. doi: 10.3390/molecules31183254 (PMC13609728; doi:10.3390/molecules31183254)
Supplement: Supplementary file 1 [file molecules-31-03254-s001.zip › molecules-4544514-supplementary.pdf]

Supporting Information

Exsolution-Engineered Perovskite Catalysts for Durable Plasma-Assisted Ammonia Synthesis

Sebastián Gámez [1], Abhyuday Chatterjee [2], Filippo Manaigo [3], Rony Snyders [2,3] and Eric M. Gaigneaux [1,*]

[1] Institute of Condensed Matter and Nanosciences (IMCN), Université Catholique de Louvain (UCLouvain), Place Louis Pasteur 1. L04.01.09, 1348 Louvain-la-Neuve, Belgium; sebastian.gamez@uclouvain.be

[2] Research Group ChIPS, Department of Chemistry, University of Mons, 20 Place du Parc, 7000 Mons, Belgium

[3] Materia Nova Research Center, 3 Avenue Copernic, 7000 Mons, Belgium; filippo.manaigo@materianova.be

* Correspondence: eric.gaigneaux@uclouvain.be

The TPR profiles show two overlapping reduction events for each perovskite precursor. The higher-temperature component, centered at 540 °C for $LaNiO_3/Al_2O_3$ and 620 °C for $LaCoO_3$/Al2O_3, is assigned to the reduction of the B-site cation (Ni^{3+} or Co^{3+}) that drives metal exsolution from the perovskite lattice, and was used to select the H_2 reduction temperature applied prior to plasma testing (550 °C for Ni, 650 °C for Co). The higher reduction temperature observed for the Co system is consistent with the greater thermal stability of the $LaCoO_3$ phase relative to $LaNiO_3$.

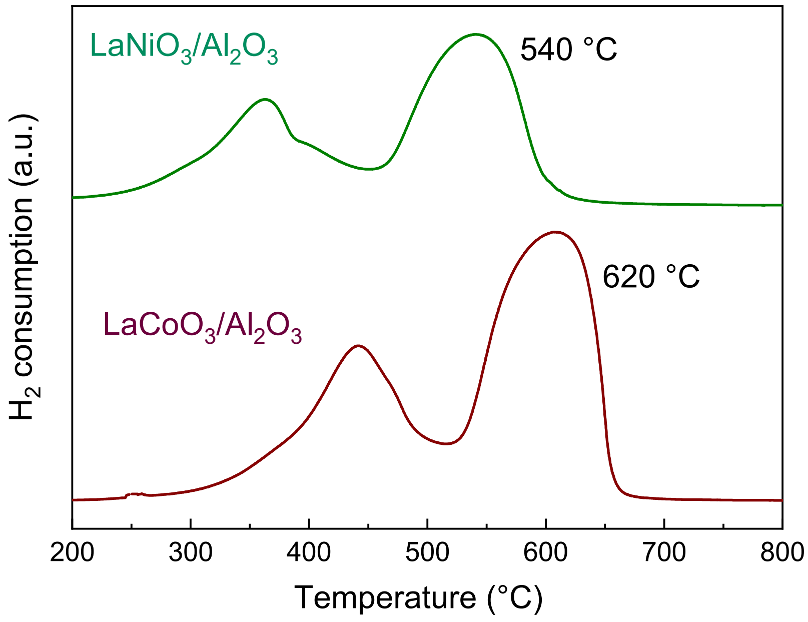

Figure S1. H_2-TPR profile of $LaNiO_3/Al_2O_3$ and $LaCoO_3/Al_2O_3$.

a) XRD pattern of the bare γ-Al_2O_3 support, showing reflections indexed to the γ- and δ-alumina phases and confirming the transition-alumina structure of the pellets prior to perovskite deposition. b) N_2 physisorption isotherm of the bare support, showing a Type IV isotherm with a well-defined hysteresis loop characteristic of the mesoporous, predominantly interparticle texture described in the main text, with a specific surface area of 232 m^2/g.

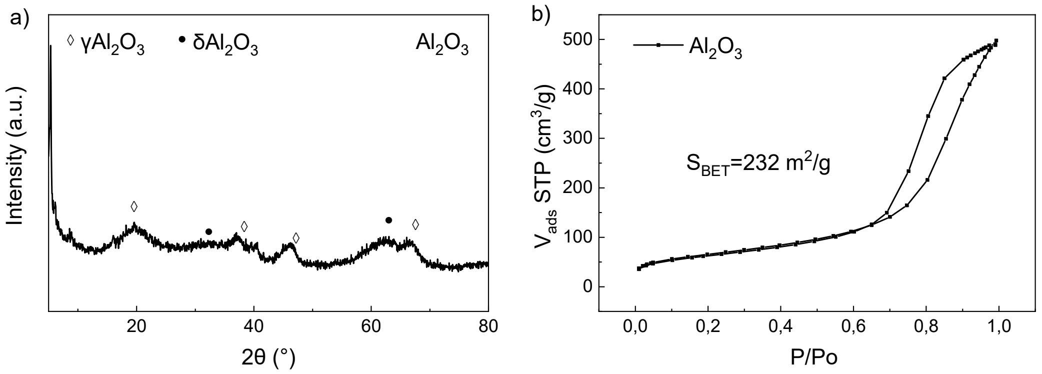

Figure S2. (a) XRD patterns and (b) N_2 physisorption isotherm of alumina pellets

Textural properties of catalysts before and after plasma catalytic conditions are summarized in Table 2:

Table S1. Textural properties of Fresh and Spent catalysts

Sample	Specific surface area (m^2/g)[a]	Pore Volume (cm^3/g)[b]	Mean Pore diameter (nm)[b]	Crystallite size (nm)[c]
Ni-La_2O_3/Al_2O_3 Fresh	83	0.21	8.5	15
Ni-La_2O_3/Al_2O_3 Spent	77	0.19	8.2	19
Co-La_2O_3/Al_2O_3 Fresh	97	0.28	8.5	13
Co-La_2O_3/Al_2O_3 Spent	93	0.27	8.4	15
Co/Al_2O_3 Fresh	200	0.61	8.2	---
Co/Al_2O_3 Spent	180	0.57	8.3	64

[a]BET method; [b]BJH method; [c]Scherrer Equation; ND: Not detected

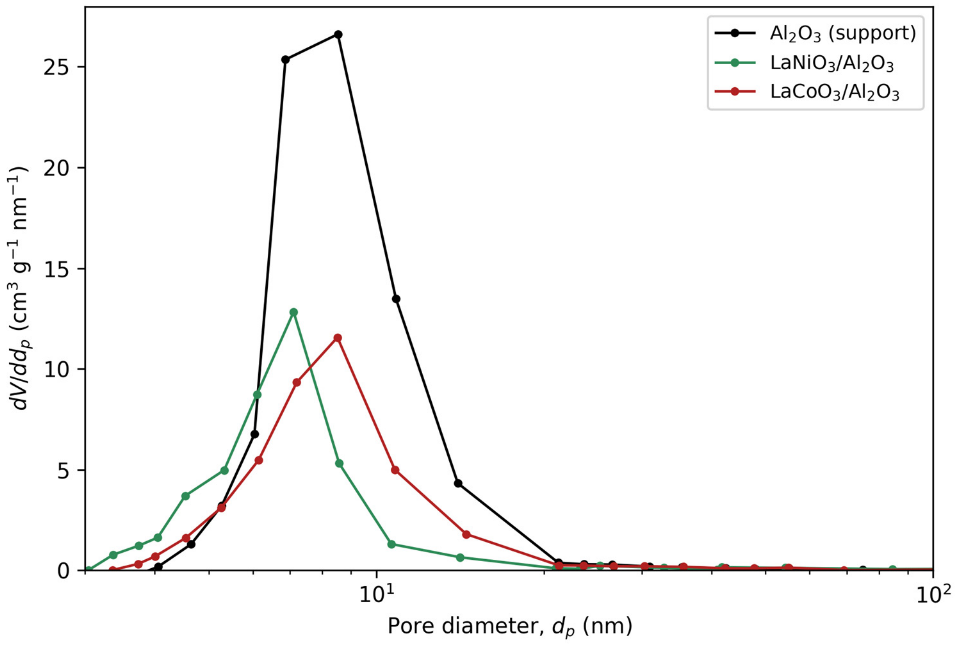

Figure S3. BJH pore size distributions (dV/dd_p) of the bare Al_2O_3 support and the $LaNiO_3/Al_2O_3$ and $LaCoO_3/Al_2O_3$ precursor materials reported in Table 1.

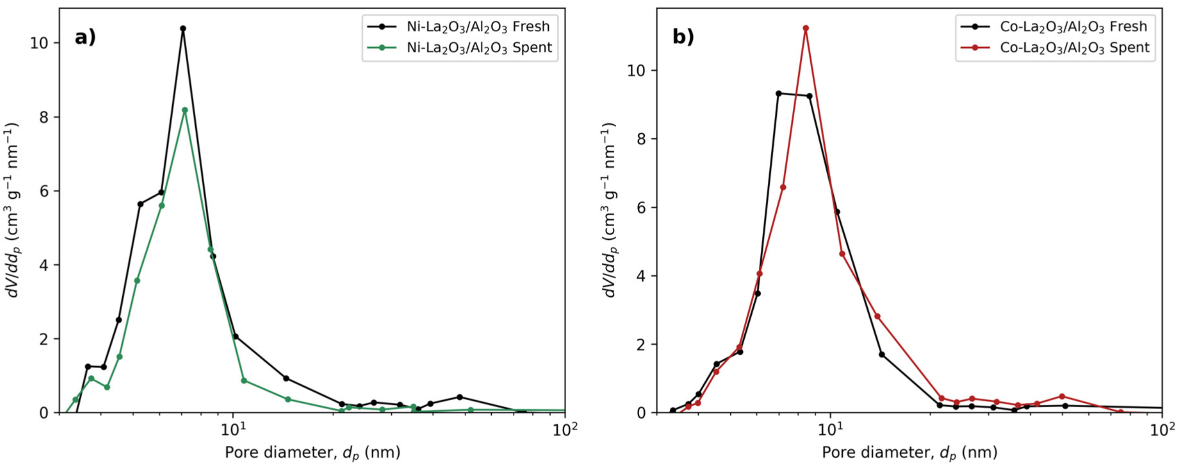

Figure S4. BJH pore size distributions (dV/dd_p) of a) $Ni-La_2O_3/Al_2O_3$ and b) $Co-La_2O_3/Al_2O_3$ catalysts, Fresh and Spent, corresponding to the mean pore diameters reported in Table S1.

Calculation of H_2 conversion and NH_3 productivity

The H_2 conversion factor and NH_3 productivity reported in the main text (Figure 3) were calculated from the downstream H_2 and NH_3 concentrations measured by gas chromatography, following the equations below.

Equation S1 (NH_3 concentration from the GC calibration curve):

$$C_{NH3}\ (\%) = 4.8708\times10^{-5} \times A_{NH3} - 2.6398\times10^{-6}$$

where A_{NH3} is the integrated GC peak area of NH_3, and the numerical coefficients are obtained from an external NH_3 calibration curve.

Equation S2 (H_2 conversion factor):

$$X(NH_3) = C_{NH3} / [C_{NH3} + (2/3)\cdot C_{H2}]$$

where C_{H2} and C_{NH3} are the measured downstream mole fractions (%) of H_2 and NH_3, respectively. This expression is algebraically equivalent to the stoichiometric form $X(H_2) = 1.5\cdot C_{NH3} / (C_{H2} + 1.5\cdot C_{NH3})$, derived from the reaction stoichiometry $N_2 + 3H_2 \rightarrow 2NH_3$ (3 mol H_2 consumed per 2 mol NH_3 formed).

Equation S3 (NH_3 productivity):

$$\text{Productivity}\ (\mu\text{mol}\ g_{cata}^{-1}\ h^{-1}) = (C_{NH3}/100) \times F_{total} \times (10^6/V_m) \times 60 / m_{cata}$$

where F_{total} is the total inlet gas flow rate (sccm), V_m = 22,414 $cm_3\ mol^{-1}$ is the standard molar volume of an ideal gas, and m_{cata} = 5 g is the catalyst mass.

Representative worked example (Co-La_2O_3/Al_2O_3, 400/400 cm^3 H_2/N_2, 0.7 kW, replicate 1):

**Parameter**	**Value**
Measured NH_3 peak area (A_{NH3})	95.14
C_{NH3} (Eq. S1)	4.631×10^{-3} %
Measured C_{H2}	0.4286 %
$X(NH_3)$ (Eq. S2)	1.60 % (cf. triplicate mean 1.63 ± 0.04 %)
Total inlet flow (F_{total})	800 sccm
NH_3 productivity (Eq. S3)	19.8 μmol g_{cata}^{-1} h^{-1} (cf. triplicate mean 20.6 ± 0.7 μmol g_{cata}^{-1} h^{-1})

H_2 conversion and NH_3 productivity

The maximum H_2 conversion (2.39%) and the maximum NH_3 productivity (20.6 μmol $gcata^{-1}$ h^{-1}) reported in the main text correspond to two different operating conditions (200/200 cm^3 H_2/N_2, 0.7 kW and 400/400 cm^3 H_2/N_2, 0.7 kW,

respectively), reflecting the conversion-throughput trade-off discussed in the main text, rather than a single paired measurement.

Table S2. Testing conditions and estimated residence time for each flow/power condition.

H_2 (cm^3/min)	N_2 (cm^3/min)	H_2:N_2 ratio	Total flow (cm^3/min)	Catalyst mass (g)	Est. residence time (ms)
200	200	1:1	400	5	5.6
200	200	1:1	400	5	5.6
400	200	2:1	600	5	5.0
400	200	2:1	600	5	4.4
400	400	1:1	800	5	4.8
400	400	1:1	800	5	4.0

Residence time was estimated as $\tau = V_{reactor} \times P_{actual} / (P_{std} \times Q_{std})$, where $V_{reactor}$ = 10.37 cm^3 (cylindrical catalyst bed, 20 mm diameter × 33 mm height), P_{actual} is the average measured reactor pressure for each condition, P_{std} = 760 Torr, and Q_{std} is the total inlet flow rate at standard conditions.
